# Expression of oestrogen and progesterone receptors in gastric cancer: a flow cytometric study

**DOI:** 10.1038/sj.bjc.6990497

**Published:** 1999-06

**Authors:** D Karat, I Brotherick, B K Shenton, D Scott, S A Raimes, S M Griffin

**Affiliations:** Northern Oesophagogastric Cancer Unit, Royal Victoria Infirmary, Newcastle upon Tyne, NE1 4LP, UK; Department of Surgery, Medical School, University of Newcastle, Newcastle upon Tyne, NE2 4HH, UK

**Keywords:** gastric cancer, oestrogen, progesterone, receptor

## Abstract

Increased expression of oestrogen (ER) and progesterone (PR) receptors have been reported in gastric adenocarcinoma, although results have been variable. Immunohistochemical staining methodologies, in particular in the detection of ER, have been inconsistent with many tumours being classified ER-negative. In this study we have used flow cytometry to quantify expression of ER and PR in gastric adenocarcinoma and examine their relationships with established prognostic indicators. Cytokeratin-positive cells obtained from tumour biopsies of 50 patients with gastric cancer and ten control patients were labelled with biotinylated ER or PR antibodies followed by streptavidin PE. Flow cytometry was seen to increase the detection of ER levels in gastric cancer with more receptor-positive patients in this study than in results published to date. We believe this is related to the sensitivity of the flow cytometric assay with the detection of small shifts in ER level detected using cytokeratin gating. On analysis, the data showed no significant correlations with tumour stage and grade, and no differences were seen between normal mucosa and gastric cancer samples. © 1999 Cancer Research Campaign


*British Journal of Cancer* (1999) **80**(8), 1271–1274
© 1999 Cancer Research Campaign
Article no. bjoc.1999.0497

### Expression of oestrogen and progesterone receptors in gastric cancer: a flow cytometric study

**D Karat[1], I Brotherick[2], BK Shenton[2], D Scott[1], SA Raimes[1] and SM Griffin[1]**

[1]Northern Oesophagogastric Cancer Unit, Royal Victoria Infirmary, Queen Victoria Road, Newcastle upon Tyne, NE1 4LP, UK; [2]Department of Surgery, Medical School, Framlington Place, University of Newcastle, Newcastle upon Tyne NE2 4HH, UK

**Summary** Increased expression of oestrogen (ER) and progesterone (PR) receptors have been reported in gastric adenocarcinoma, although results have been variable. Immunohistochemical staining methodologies, in particular in the detection of ER, have been inconsistent with many tumours being classified ER-negative. In this study we have used flow cytometry to quantify expression of ER and PR in gastric adenocarcinoma and examine their relationships with established prognostic indicators. Cytokeratin-positive cells obtained from tumour biopsies of 50 patients with gastric cancer and ten control patients were labelled with biotinylated ER or PR antibodies followed by streptavidin PE. Flow cytometry was seen to increase the detection of ER levels in gastric cancer with more receptor-positive patients in this study than in results published to date. We believe this is related to the sensitivity of the flow cytometric assay with the detection of small shifts in ER level detected using cytokeratin gating. On analysis, the data showed no significant correlations with tumour stage and grade, and no differences were seen between normal mucosa and gastric cancer samples.

**Keywords**: gastric cancer; oestrogen; progesterone; receptor

Binding of hormones to their receptors results in formation of stabilized complexes which interact with specific regions of DNA (Yamamato and Alberts, 1976). This leads to increased transcription of hormone-dependant genes, translation into proteins, and eventually replication and tumour cell division and growth.

Receptors for sex hormones have been identified in several ‘hormone-dependent’ organs such as breast, endometrium and prostate (Smith et al, 1975; Walsh and Hicks, 1979; Howell et al, 1984). The presence of hormone receptors has been found to correlate with a number of clinicopathological factors and may be of prognostic significance. Furthermore, endocrine manipulation plays an important role in the treatment of these malignancies. More recently, receptors for sex hormones, particularly those for oestrogen, have been described in ‘non-hormone-dependent’ tumours, such as those of the colon, kidney, pancreas and liver (McClendon et al, 1977; Kune and Hunt, 1984; Kohigashi et al, 1987). Oestrogen (ER) and progesterone receptors (PR) have been detected in adenocarcinoma of the stomach (Tokunaga et al, 1986). Endocrine manipulation can play an important part in the treatment of many of these malignancies and gives supportive evidence to the role of sex hormone receptors in cancer.

The significance of ER and PR in gastric cancer has yet to be determined. In vitro and in vivo studies have yielded conflicting data as to the effect of oestradiol on gastric cancer (Furukawa et al, 1982; Nohga et al, 1987; Harrison et al, 1989*a*). The prognostic significance of receptor expression is the subject of conflicting reports and remains unclear (Tokunaga et al, 1986; Yozozaki et al, 1988; Harrison et al, 1991). Clinical studies of the efficacy of adjuvant endocrine therapy, mainly involving the administration of tamoxifen, have also been inconclusive, and many studies too small and poorly controlled (Kitaoka, 1983; Kojima and Takahashi, 1986; Harrison et al, 1989*b*). Nevertheless, determination of sex hormone receptor expression in gastric cancer may play an important role in the understanding of the biochemical and pathophysiological behaviour of this disease.

Conventional assays for ER and PR have relied on biochemical techniques such as dextran-coated charcoal assay, immunohistochemistry (using polyclonal and, more recently, monoclonal antibodies), enzyme immunoassay and radio-ligand binding assay. The enormous variation in receptor level expression demonstrated by these assays has consequently shown ranges of between 0% and 67% of gastric cancers are positive for ER (Wu et al, 1992*a*; Ismail et al, 1994) and between 9% and 83% are positive for PR (Sica et al, 1984; Wu et al, 1992*a*).

Use of a flow cytometric assay to quantify receptors in gastric cancer remains relatively untried. Use of this technique has been developed for the analysis of breast cancer where it has shown excellent correlation with the radioligand binding assay (Brotherick et al, 1994).

The aim of this prospective study was to accurately quantify the expression of ER and PR expression in gastric cancer, as well as normal gastric mucosa, using two-colour flow cytometry. We have further examined the relationship between receptor expression and established prognostic indicators.

### MATERIALS AND METHODS

Fifty patients with histologically proven gastric cancer were recruited for this study. Tissue for analysis was obtained from either endoscopic biopsies of the gastric tumour or gastrectomy specimen obtained immediately following surgical resection. A further ten biopsies were obtained from patients undergoing routine endoscopy and confirmed as histologically ‘normal’.

*Received 30 April 1998*
*Revised 14 October 1998*
*Accepted 22 January 1999*

*Correspondence to:* D Karat

Tissues were snap-frozen in liquid nitrogen at –70°C and stored in liquid nitrogen until required. Standard patient and routine pathological data (including tumour grade and stage) were collected prospectively and stored on a database.

### ER and PR standardization with flow cytometry

The method described by Brotherick et al (1994, 1995*a*) was used. Incubation of Quantum Simply Cellular beads (QSC, Flow Cytometry Standards Corp., NC, USA) with an excess of ER- and PR-conjugated antibody was performed to saturate all binding sites. The beads were washed with Isoton II (Coulter) and analysed by flow cytometry on a FACScan flow cytometer (BD) and analysed with Lysis II software (Becton Dickinson). Four binding capacity peaks and one blank peak for non-specific binding were seen on the FL1 histogram. The median fluorescence channel for each peak was taken, and a regression curve of linear channel number against binding capacity was constructed and the equation for the line calculated with Quickcal QSC calibration software with calibration for non-specific binding. Conversion of known linear fluorescence channel numbers into binding capacities for cells labelled with ER or PR could then be performed.

### Preparation of cells for flow cytometry

Samples of gastric tissue were finely minced and further disaggregated by passing through a fine wire mesh (50 µm) to form a single cell suspension. The resulting cell suspension was centrifuged at 400 ***g*** for 10 min and the resulting pellet resuspended at a concentration of approximately $1 \times 10^6$ cells $ml^{-1}$ lsoton II (Coulter, Luton, UK). Fifty microlitres of cell suspension were aliquotted into LP10 tubes (SH Scientific, Northumberland, UK). To each sample, 50 µl of 2% saponin (BDH, in Isoton II) was added with gentle mixing. Six tubes were prepared for each sample as follows: (1) unstained control; (2) 2 µl of mouse IgG-2b FITC isotype control (Coulter); (3) 10 µl of streptavidin–phycoerythrin only (SA-PE control; Becton Dickinson, Oxford, UK); (4) 2 µl of anti-cytokeratin LP-34 (DAKO AS, Denmark); (5) 2 µl of LP-34 antibody plus 2 µl of anti-ER antibody (ER1D5 biotin-conjugated, DAKO); (6) 2 µl of LP-34 antibody plus 2 µl anti-PR antibody (NCL-PR, biotin-conjugated; Novocastra, Newcastle upon Tyne, UK). All samples were mixed, incubated at 4°C for 20 min and then washed with Isoton II containing 1% saponin. To those cells labelled with biotinylated antibody, 10 µl SA-PE (BD) was added to the cell pellet as previously described. After incubation and washing the cell pellet was resuspended in 0.5 ml Isoton II, and flow cytometry was performed on a FACScan flow cytometer (BD) using prestored settings.

### Analysis by flow cytometry

Data analysis was performed using Lysis II software. Cellular debris was excluded by a live gate (R1) set on a dot plot of forward scatter light (FSC) against side scatter (SSC). A minimum of 10 000 events were collected in the gate R1. Cytokeratin-positive (LP34-positive) cells were further gated (R2) and median fluorescence (PE) values determined from the FL2 histogram for SA-PE-stained (control), ER- and PR-stained cells. Binding capacities for the cells were calculated from the standardized QSC bead equation as described above, and receptor status was therefore evaluated as the number of binding sites per cell. A cut-off value of 3000 sites per cell was used, with numbers below this regarded as negative for the receptors. This was determined using earlier published work comparing the flow cytometric assay with radio-ligand binding assays in breast carcinoma (Brotherick et al, 1994).

### Consistency of staining

A stock solution of gastric cancer cell line AGS (positive for ER and PR) and fresh lymphocytes (a negative control) were stained using the method above on three occasions for ER and PR. Intra-assay reproducibility was confirmed.

### Statistical analysis

Statistical analysis was performed using Minitab (release 9.2) software. Receptor expression for the groups analysed were expressed as a median value with 95% confidence intervals (CI). Groups were compared using Mann–Whitney *U*-test and significance assumed for a *P*-value less than 0.05. Data were displayed as box plots.

### RESULTS

Fifty gastric cancers and ten histologically ‘normal’ stomachs were analysed by flow cytometry. The mean (s.e.m.) age of the patients with gastric cancer was 67.9 (1.23) years, and of the patients with histologically ‘normal’ stomachs was 65.5 (2.04) years. Of the patients with gastric cancers, 32 were male and 18 female compared with six histologically normal males and four females.

### Hormone receptor expression in tumours vs normal stomach

Thirty-six tumours (72%) expressed ER compared with seven (70%) histological ‘normals’. PR were expressed in 47 (94%) of the tumours and nine (90%) of the ‘normals’. Only two tumours and one ‘normal’ expressed neither ER nor PR. There were no significant differences in ER expression on comparing gastric cancer and ‘normal’ gastric mucosa groups (medians = 8505 and 9529 respectively; CI 7794 to 5976; $P = 0.808$). The same was true for PR (medians = 40 552 and 29 331; CI 51 308 to 16 000; $P = 0.496$).

### Sex difference

Twenty-two tumours from male patients (69%) expressed ER compared with 14 (78%) from females. Thirty-one tumours from males (97%) expressed PR compared with 16 (89%) from females. No significant differences were determined on comparison of ER (male vs female medians = 10 245 and 6194; CI 5927–7889; $P = 0.977$) or PR (male vs female medians = 42 602 and 34 915; CI 29 104–33 515; $P = 0.718$) receptor levels.

### Grade and stage

Due to the relatively small numbers, grading was limited to two groups: well-differentiated (including moderately differentiated tumours) and poorly differentiated (including undifferentiated tumours). All histological grading was performed by a single consultant histopathologist using standard histological criteria.

 *© 1999 Cancer Research Campaign*

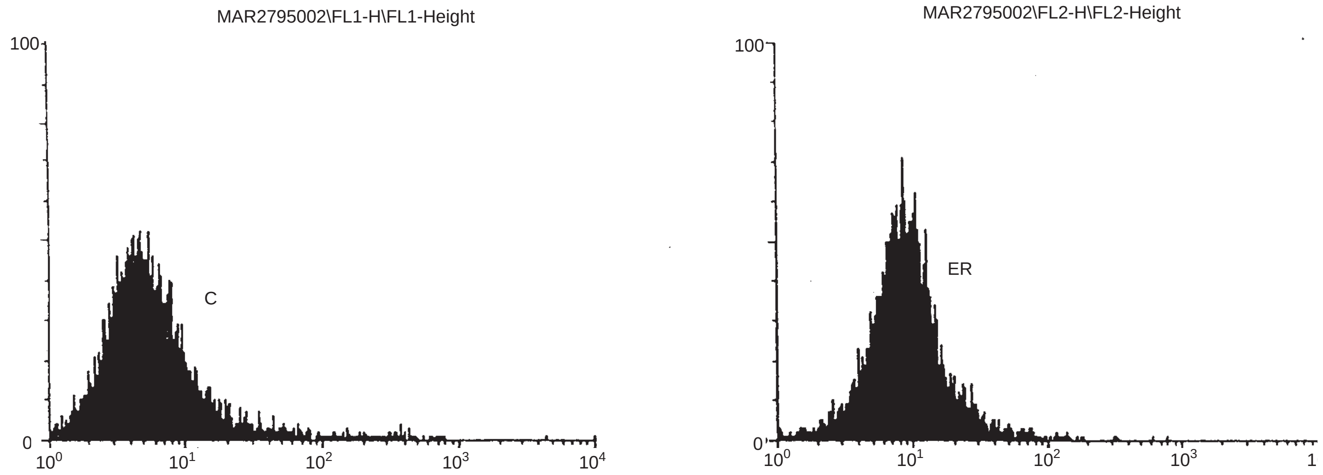

**Figure 1** Fluorescence histograms representing staining for cytokeratin (C) and oestrogen receptors (ER). This sample was stained for ER only and shows a very low median fluorescence due to the inclusion of non-epithelial cells and debris

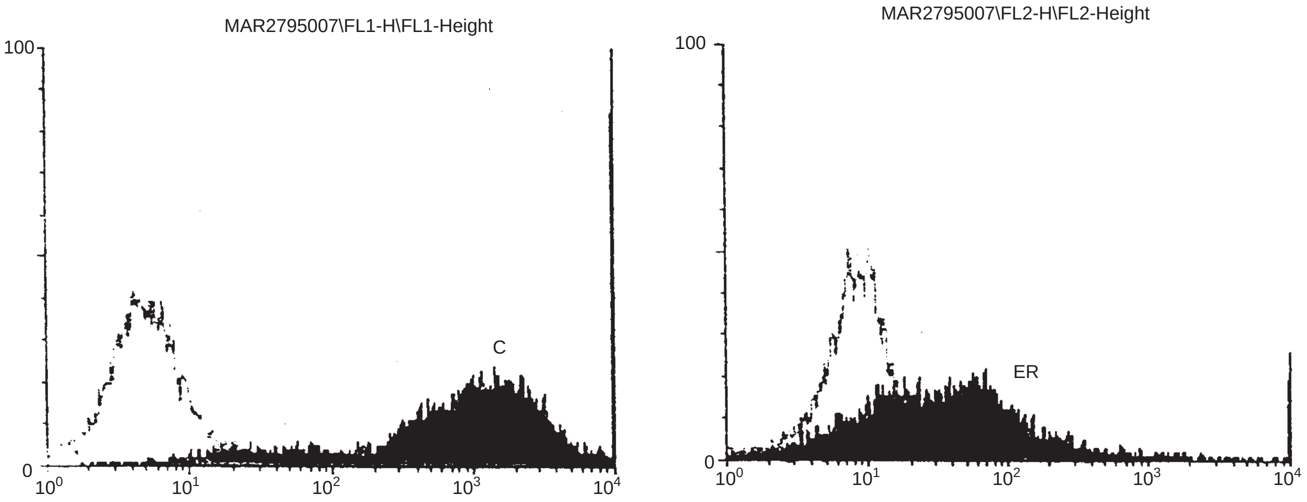

**Figure 2** Fluorescence histograms representing staining for cytokeratin (C) and oestrogen receptors (ER) as before, on the same population of cells. However, this sample was stained for both ER and C. The addition of a 'cytokeratin gate' results in a significant increase in the median fluorescence value (shift of peak to the right) for ER by the inclusion of only cytokeratin-positive (epithelial-derived) cells and the exclusion of other cells and debris

Staging followed standard TNM classification for gastric cancer for the 'T' stage, while the 'N' stage was simplified to node-negative (NO) or -positive (N1, N2).

Sixteen tumours were graded as well-differentiated and 34 as poorly differentiated. Seven tumours were stage T1, four were T2, 23 were T3 and 16 were T4. Thirty-eight tumours had lymph node metastases, while 12 were node-negative.

No significant differences were demonstrated for ER expression with respect to tumour grade (well vs poor; medians = 6569 and 9055; CI 8505 to 5519; $P$ = 0.633) or stage. Similarly, expression of PR with respect to tumour grade (well vs poor; medians = 49 292 and 38 669; CI 32 371 to 32 552; $P$ = 0.649;) and stage showed no significant difference.

### DISCUSSION

Sex hormone receptor status has been reported to be of prognostic value in gastric cancer, although more recently its value with respect to both prognosis and therapeutic response to endocrine therapy has been disputed as already discussed. Conventional assays for both ER and PR have been inconsistent and subject to the limitations of the technique used. Cohen et al (1988) described the use of image cytometry to allow both quantitation and examination of heterogeneous tumours. We reported the use of ER 1D5 antibody in conjunction with anti-cytokeratin antibody to quantify the ER status of primary breast cancers by flow cytometry without the need for fixation and prolonged incubation (Brotherick et al, 1995*b*).

In this study we have applied this flow cytometric methodology to the examination of gastric tumour and normal mucosa. We have demonstrated ER and PR in a greater proportion of gastric cancers than published to date. Two-colour flow cytometry has already proven to be an accurate assay for the quantification of ER in breast cancer using the DAKO ER1D5 biotinylated antibody (Brotherick et al, 1995*a*). Use of a cytokeratin 'gate' increases the assay sensitivity as shown in Figures 1 and 2, by exclusion of non-epithelial cells and debris. The technique has been reported to be an accurate and quantitative method for determining receptor

*© 1999 Cancer Research Campaign* 

expression on tumour cells (Brotherick et al, 1995*b*) and therefore offers advantages over immunohistochemistry. In this study we have shown that the rapid, simple method for hormone receptor assay can be applied to small biopsies, such as those from preoperative endoscopy. This may be of importance if hormone manipulation forms a part of the adjuvant treatment of gastric cancer.

Our findings support those of Wu et al (1992*a*), finding no significant difference in ER or PR expression in gastric cancer compared with normal gastric mucosa using DCC and EIA assays. This is in contrast to the data reported by Kojima et al (1991) which demonstrated expression of ER in gastric cancer, but not in normal mucosa when measured by immunohistochemistry and recent data from Singh et al (1997) showing decreased expression of ER in gastric cancer compared to normal mucosa. Our data showed no significant difference between receptor-positive and receptor-negative groups on comparison with sex, tumour grade and tumour stage. Similarly, the presence or absence of sex hormone receptors showed no correlation with the prognostic indicators studied.

On the basis of these results, expression of sex hormone receptors in gastric cancer is of little clinical significance. The role of hormonal therapies remains unclear despite the finding that limited (and often uncontrolled) studies have shown benefit with tamoxifen (Kitaoka, 1983; Kojima and Takahashi, 1986). Our findings would, however, complement the findings of a large British study which failed to show any benefit from tamoxifen therapy (Harrison et al, 1989*b*). However, the same study also identified the presence of ER to be an independent prognostic factor in gastric cancer, and using immunohistochemistry to examine receptor status, they reported 55.8% of tumours positive compared to 70% in our study. These discrepancies may well be explained by the small patient numbers in this study in combination with a more sensitive method of receptor quantification.

In summary, this study found no significant difference in sex hormone expression on comparing gastric cancer and normal mucosa. No correlation between receptor expression and known prognostic indicators was found. While sex hormone receptors may play a biological role in tumour growth and development, routine assay for ER and PR in gastric cancer is at present of no clinical benefit in the management of the disease.

### ACKNOWLEDGEMENTS

The authors would like to thank Marianne Broe of DAKO A/S Denmark for the provision of conjugated ER-1D5, her cooperation throughout this study and her continued support. We would also like to thank the Northern and Yorkshire Regional Health Authority (Research and Development) for their financial support.

### REFERENCES

Brotherick I, Lennard TWJ, Wilkinson SE, Cook S, Angus B and Shenton BK (1994) A flow cytometric method for the measurement of epidermal growth factor receptor: a comparison with the radio-ligand binding assay. *Cytometry* **16**: 262–269

Brotherick I, Lennard TWJ, Cook S, Johnstone R, Angus B, Winthereik MP and Shenton BK (1995*a*) Use of the biotinylated antibody DAKO-ER 1D5 to measure oestrogen receptor on cytokeratin positive cells obtained from primary breast cancer cells. *Cytometry* **20**: 74–80

Brotherick I, Shenton BK and Lennard TWJ (1995*b*) Are fine-needle aspirates representative of the underlying solid tumour? A comparison of receptor levels, ploidy, and the influence of cytokeratin gates. *Br J Cancer* **72**: 732–737

Cohen O, Brugal G, Seigneurin D and Demongeot J (1988) Image cytometry of estrogen receptors in breast carcinomas. *Cytometry* **9**: 579–587

Furukawa H, Iwanaga T, Koyama H and Taniguchi H (1982) Effect of sex hormone on carcinogenisis in the stomach of rats. *Cancer Res* **42**: 5181–5182

Harrison JD, Watson S and Morris DL (1989*a*) The effect of oestradiol and tamoxifen on the growth of human gastric cancer cell lines. *Cancer* **63**: 2148–2151.

Harrison JD, Morris DL, Ellis IO, Jones JA and Jackson I (1989*b*) The effect of Tamoxifen and estrogen receptor status on survival in gastric carcinoma. *Cancer* **64**: 1007–1010.

Harrison JD, Jones JA, Ellis IO and Morris DL (1991) Oestrogen receptor D5 antibody is an independent negative prognostic factor in gastric cancer. *Br J Surg* **78**: 334–336.

Howell A, Barnes DM, Harland RN, Redford J, Bramwell VH and Wilkinson MJ (1984) Steroid hormone receptors and survival after first relapse in breast cancer. *Lancet* **1**: 588–591.

Ismail T, Baker P, Hale C, Fielding JWL and Hallissey MT (1994) Oestrogen receptors in gastric cancer: international differences. *Eur J Surg Oncol* **20**: 512

Kitaoka H (1983) Sex hormone dependency and endocrine therapy for patients with diffuse carcinoma of the stomach. *Jpn J Cancer Chemother* **10**: 2453–2460

Kohigashi K, Fukuda Y and Imura H (1987) Estrogen receptors in hepatocellular carcinoma: is endocrine therapy for hepatocellular carcinoma likely to be effective? *Gastroenterol Jpn* **22**: 322–330

Kojima O and Takahashi T (1986) Endocrine therapy for scirrhous carcinoma of the stomach. *Jpn J Cancer Chemother* **13**: 2526–2531

Kojima O, Takahashi T, Kawakami S, Uehara Y and Matsui M (1991) Localisation of estrogen receptors in gastric cancer using immunohistochemical staining of monoclonal antibody. *Cancer* **67**: 2401–2406

Kune GA and Hunt RF (1984) Oestrogen receptors in cystadenocarcinoma of the pancreas. *Aust NZ J Surg* **54**: 321–323

McClendon JE, Appleby D, Claudon DB, Donegan WL and Decosse JJ (1977) Colonic neoplasms: tissue estrogen receptor and carcinoembryonic antigen. *Arch Surg* **122**: 240–241

Nogha K, Katsumata T and Ishiwata K (1987) Experimental and clinical study on chemoendocrine therapy for gastric cancer. *J Jpn Surg Soc* **88**: 1109–1112

Sica V, Nola E, Contieri E, Bova R, Masucci MT, Medici N, Petrillo A, Weisz A, Molinari AM and Puca GA (1984) Estradiol and progesterone receptors in malignant gastrointestinal tumours. *Cancer Res* **44**: 4670–4674

Singh S, Poulsom R, Wright NA, Sheppard MC and Langman MJS (1997) Differential expression of oestrogen receptor and oestrogen inducible genes in gastric mucosa and cancer. *Gut* **40**: 516–520

Smith DE, Prentice R, Thompson DJ and Herrman WL (1975) Association of exogenous estrogen and endometrial carcinoma. *N Engl J Med* **293**: 1164–1167.

Tokunaga A, Nishi K, Matsukura N, Tanaka N, Onda M, Shirota A, Asano G and Hayashi K (1986) Estrogen and progesterone receptors in gastric cancer, *Cancer* **56**: 1376–1379

Walsh PC and Hicks LL (1979) Characterisation and measurement of androgen receptors in human prostatic tissue. *Prog Clin Biol Res* **33**: 51–63

Wu CW, Chang HM, Hsieh MC, Kao HL, Lui WY, P'eng FK and Chi CW (1992*a*) The nontransformed progesterone and estrogen receptors in gastric cancer. *Gastroenterology* **102**: 1639–1646

Wu CW, Tsay SH, Chang TJ, Chang HM, Hsieh MC, Lui WY, P'eng FK and Chi CW (1992*b*) Clinicopathologic comparisons between estrogen receptor-positive and –negative gastric cancers. *J Surg Oncol* **51**: 231–235

Yamamoto KR and Alberts BM Steroid receptors: elements for modulation of eukaryotic transcription. *Ann Rev Bioch* **45**: 721–746.

Yozozaki H, Takekura N, Takanashi A, Tabuchi J, Haruta R and Tahara E (1988) Estrogen receptors in gastric adenocarcinoma: a retrospective immunohistochemical analysis. *Virchows Archiv [A]* **413**: 297–302

 © 1999 Cancer Research Campaign
